# Editorial: Theory of Mind in Humans and in Machines

**DOI:** 10.3389/frai.2022.917565

**Published:** 2022-05-12

**Authors:** Christelle Langley, Bogdan-Ionuţ Cîrstea, Fabio Cuzzolin, Barbara Jacquelyn Sahakian

**Affiliations:** ^1^Department of Psychiatry, School of Clinical Medicine, University of Cambridge, Cambridge, United Kingdom; ^2^School of Engineering, Computing and Mathematics, Oxford Brookes University, Oxford, United Kingdom

**Keywords:** human theory of mind, machine theory of mind, human-robot interaction, cognitive science and neuroscience, artificial intelligence

Humans can think flexibly and creatively, whereas current artificial intelligence (AI) systems may fail to recognize instances where they would need to change the solution approach, when the current approach is unsuccessful. For a long time, AI systems used to be incapable of matching human capabilities for the vast majority of tasks. However, this has recently started to change. For example, AlphaZero is able to generate new solutions in chess and successfully outperform even Chess Grandmasters (Sadler and Regan, 2019). Similarly, initially robotics were bulky and unrefined and unable to perform even basic human actions, such as picking up a glass. More recently there has been significant progress. The Shadow Robot Company, together with the Human Brain Project, have now created the world's first haptic telerobot hand that has very similar movements to those of humans (Sahakian et al., 2021). Similarly to computers, which used to take up a lot of space and were cumbersome to use but are now in our mobile phones and usable by anyone, AI systems are likely to become much more widely used and more impactful on a societal level. For example, some of the more recent AI breakthroughs could, in the long-run, have significant impacts in fields like drug design (e.g., AlphaFold; Jumper et al., 2021), software engineering (e.g., Codex; Chen et al., 2021) or graphic design, through image generation (e.g., DALL-E 2; Ramesh et al., 2022). Even in the near future, we can expect at least some types of AI systems (e.g., autonomous cars) to become commonplace in society. The expected wider societal impacts of AI systems makes it even more important to ensure that they will be used to promote wellbeing in individuals, enhance innovation and to benefit society. Therefore, it is important to consider ethical issues associated with human-AI interactions, including components of social cognition, such as empathy and Theory of Mind (ToM) and their integration into AI systems (Cuzzolin et al., 2020).

ToM is the ability of the human mind to attribute mental states to others and is a key component of human cognition. ToM encompasses inferring others' beliefs, desires, goals, and preferences. The same capability of inferring human mental states is a prerequisite for AI to be integrated into human society. Aspects related to ToM have been an area of interest in User Modeling, Student / Learner Modeling, User-Adaptive Systems Design, and Personalization. All of these related areas have been using AI techniques for determining users' goals and interests to adapt interactive software systems to their human users' needs.

This Research Topic aimed to span across the fields of artificial intelligence, cognitive science, and neuroscience with the intention to formulate computational proposals of cognitive science and neuroscience-inspired ToM. The intention was to allow for the comparison of the strengths and limitations of ToM, Inverse Reinforcement Learning, and other reward specification methods and to establish common baselines, metrics, and benchmarks, and to identify open questions.

Each of the following five articles made a novel and complementary contribution to this Research Topic.

Schellen et al. investigated deception in human-robot interaction, focusing on the effect that eye contact with a robot has on honesty toward a robot. Their results showed that humans are more honest after a robot establishes eye contact with them. However, this is only true in response to deceptive, but not honest behavior. These results suggest that robots can be perceived and treated as social agents in a similar way to human-human interactions.

Nakahashi and Yamada showed that, in a human-AI collaborative setting, the AI providing implicit guidance to the human can create a balance between improving the human's plans and maintaining the human's autonomy in a way that preserves their autonomy better than explicit guidance. The implicit guidance provided is based on the ToM capability that humans can infer others' intentions based on their behavior.

Gros proposed that emotions, rather than being a leftover of a more primitive heritage, serve as a mechanism for attributing values to behavioral options. He introduced a framework in which an agent's timeline of experienced emotions is compared with the agent's “character”, defined as a preferred distribution over emotional states. In this framework, the agent's long-term goal is to choose individual tasks so that their emotional experience is aligned with their character.

Williams et al. discussed how ToM in AI is crucial for an agent's ability to interact with human team members. They suggested that, for an artificial agent to effectively engage in human-robot interactions, it must possess elements of ToM to perceive, interpret, and generate combinations of social cues. Interestingly, the authors suggested that an interdisciplinary approach is required to achieve such a model.

Langley et al. provided a review which aimed to synthesize the current knowledge about human ToM, from cognitive science and neuroscience, and machine ToM, from AI. They suggested that as yet AI (unlike human ToM) has not provided a truly holistic approach to ToM, and has rather focused on separate components. The authors stated that they intend to stimulate discussion to better integrate the fields of cognitive neuroscience and AI, particularly with regards to integrating ToM in machines.

Predictions about the future are inherently uncertain, so it is hard to make very confident statements about what AI systems will ultimately be capable of or what kind of AI approaches might help bridge the gap to human capabilities. Despite this, we think it is likely that a better integration of ToM findings from cognitive neuroscience and AI will be useful, even if it were only to help improve Human-AI interaction. If we want AI systems to be capable of inferring and motivated to respect human preferences, how humans do ToM seems an obvious place to look for ideas. We think the stakes are likely to only get higher.

## Author Contributions

CL and BC contributed equally to the manuscript. FC and BJS contributed equally to the manuscript. All authors listed have made a substantial, direct, and intellectual contribution to the work and approved it for publication.

## Funding

This work was funded by a Leverhulme Trust grant to FC and BJS under the Research Programme Grant (RPG-2019-243). CL and BC were funded by the Leverhulme Trust (RPG-2019-243).

## Conflict of Interest

FC is the Director of Oxford AI Consulting, and does consultancy for Huawei Canada. BJS consults for Cambridge Cognition. The remaining authors declare that the research was conducted in the absence of any commercial or financial relationships that could be construed as a potential conflict of interest.

## Publisher's Note

All claims expressed in this article are solely those of the authors and do not necessarily represent those of their affiliated organizations, or those of the publisher, the editors and the reviewers. Any product that may be evaluated in this article, or claim that may be made by its manufacturer, is not guaranteed or endorsed by the publisher.
